# An Emergency Department Case of Prinzmeta Angina: A Story in Pictures

**DOI:** 10.51894/001c.165603

**Published:** 2026-07-31

**Authors:** Riteesh Gajjela, Jasmine Philipose, Alex Hinken, Elizabeth Shaheen

**Affiliations:** 1 Henry Ford - Wyandotte

## 83

### Background

Prinzmetal angina, or vasospastic angina, is a reversible cause of ST-segment elevation that can closely mimic STEMI. While transient ischemic ECG changes are well documented, high-grade atrioventricular block during active coronary spasm is less commonly observed in real time. Prompt recognition in the ED is essential, as management and disposition differ from plaque-mediated acute coronary syndromes.

### Case Presentation

A 50-year-old woman with hypertension, type 2 diabetes, hyperlipidemia, tobacco use, and prior angina presented with intermittent chest pain radiating to her left arm, diaphoresis, and shortness of breath. Initial ECG revealed ST-segment elevations in inferior and anterolateral leads, prompting STEMI activation. During ED observation, she experienced ongoing chest pain. Continuous telemetry captured dynamic ST-segment elevations with transient third-degree AV block. She received chewable aspirin, morphine, and heparin. After symptom resolution, repeat ECG showed normal sinus rhythm without ischemic changes or AV block, highlighting the rapid reversibility of vasospasm-induced conduction disturbances. Emergent coronary angiography revealed severe coronary vasospasm without obstructive coronary artery disease. Intracoronary nitroglycerin resolved the spasm, normalizing ST segments and AV conduction. Laboratory studies showed mildly elevated troponin, consistent with demand ischemia. During hospitalization, she developed episodic torsades de pointes, requiring intensive cardiac monitoring.

### Management and Disposition

She was started on CCBs and long-acting nitrates per vasospastic angina guidelines. Electrophysiology consultation was obtained due to malignant arrhythmias. She was discharged with a wearable cardioverter-defibrillator for secondary prevention, with consideration for permanent ICD placement and structured outpatient follow-up.

### Discussion

This case highlights the diagnostic challenge of vasospastic angina presenting as a STEMI mimic in the ED. Emergency physicians must initially treat such presentations as acute coronary occlusion while recognizing that transient coronary vasospasm can produce dramatic but reversible ECG changes, including high-grade AV block. Continuous telemetry and serial ECGs in the ED can capture dynamic ischemic patterns that aid diagnosis. Early analgesia and nitrate therapy may rapidly reverse vasospasm and associated conduction abnormalities, while prompt cardiology consultation facilitates definitive evaluation. Recognition of Prinzmetal angina allows transition from STEMI protocols to targeted therapy with calcium channel blockers and nitrates while ensuring monitoring for malignant arrhythmias.

